# An Inflammatory Cytokine Milieu is Prominent in Premalignant Oral Lesions, but Subsides when Lesions Progress to Squamous Cell Carcinoma

**DOI:** 10.4172/2155-9899.1000230

**Published:** 2014-06-27

**Authors:** Danielle Woodford, Sara D Johnson, Anna-Maria A De Costa, M Rita I Young

**Affiliations:** 1 Research Service, Ralph H. Johnson Veterans Affairs Medical Center, Charleston, SC, USA; 2 Department of Otolaryngology, Head and Neck Surgery, Medical University of South Carolina, Charleston, SC, USA; 3 Hollings Cancer Center, Medical University of South Carolina, Charleston, SC, USA

**Keywords:** Cytokines, HNSCC, IL-17, IL-23, Inflammation, Oral cancer, Premalignant lesions

## Abstract

While head and neck squamous cell carcinomas (HNSCC) are associated with profound immune suppression, less is known about the immunological milieu of premalignant oral lesions. The present study shows dynamic shifts in the immune milieu within premalignant oral lesions and when they have progressed to HNSCC. Specifically, this study showed that the premalignant lesion environment consists of inflammatory mediators and IL-17, but this inflammatory phenotype declines when premalignant oral lesions have progressed to HNSCC. The cytokine profiles of human tissues did not correspond with plasma cytokine profiles. A murine carcinogen-induced premalignant lesion model that progresses to HNSCC was used to examine cytokine profiles released from tissues as well as regional lymph nodes. As in human tissues, murine premalignant lesions and regional lymph nodes released high levels of inflammatory cytokines and, very prominently, IL-17. Also similar to human tissues, release of inflammatory cytokines declined in HNSCC tissues of mice and in the regional lymph nodes of mice with HNSCC. Studies focusing on IL-17 showed that mediators from premalignant lesions stimulated normal spleen cells to produce increased levels of IL-17, while mediators from HNSCC were less stimulatory toward IL-17 production. IL-17 production by Th17-skewed CD4+ cells was strongly inhibited by normal oral epithelium as well as HNSCC. In contrast, premalignant lesion-derived mediators further increased IL-17 production by Th17-skewed cells. The stimulation of IL-17 production by premalignant lesions was dependent on IL-23, which premalignant lesions released in higher amounts than control tissues or HNSCC. HNSCC tissues instead produced increased levels of TGF-β compared to premalignant lesions, and skewed normal spleen cells toward the Treg phenotype. This skewing was blocked by supplementation with IL-23. These studies suggest IL-23 to be a significant contributor to the inflammatory IL-17 phenotype in premalignant oral lesions and suggest the decline in IL-23 in HNSCC leads to a decline in Th17 cells.

## Introduction

Despite advances in diagnosis and treatment, the 5-year survival rates for patients with head and neck squamous cell carcinoma (HNSCC) have remained at only 60% [1]. HNSCC is a highly aggressive disease that develops from the oral epithelium and its appearance is often preceded by premalignant oral lesions, most commonly leukoplakias [2]. Even with advanced treatments for these lesions, about 30% progress to cancer [3].

Among the hallmarks of HNSCC is the profound inhibition of immune competence that is mediated in part by immunosuppressive cell populations including immune inhibitory macrophages, T-cells that are skewed toward a Th2 phenotype, Treg cells, myeloid-derived suppressor cells (MDSC) and the less mature CD34^+^ progenitor cells [4–6]. While systemic immune suppression is prominent in patients with HNSCC, immune alterations during the premalignant stage have not been well defined. Our prior study in a carcinogen-induced oral premalignant lesion mouse model showed an increased percentage of conventional T-cells and cells expressing markers for activation, memory, and exhaustion compared to either healthy control or HNSCC-bearing mice [7]. In addition, these studies showed the premalignant stage to be associated with robust immune reactivity involving increases in inflammatory, Th1/Tc1 and Th17 cells in the regional lymph nodes, while the number of Treg cells increased prominently in mice with HNSCC. Similar to our studies with the mouse premalignant oral lesion model, Th17 levels have been shown to be increased in patients with gastric and ovarian cancers, with a shift from Th17 to Treg cells in advanced disease [8,9]. There is literature implicating IL-17 signaling as being pro-tumorigenic such as in promoting pancreatic neoplasia [10]. Inflammation has also been shown to induce immune inhibitory cells such as MDSC [11,12]. Th17 cell-associated inflammation has been shown to promote the initiation and early growth of tumors in several different cancer models [13,14]. However, other studies have shown a contrasting role of inflammation and Th17 cells in the cancer setting. For example, an increased presence of Th17 cell has been associated with improved prognosis in early stage ovarian cancer and malignant pleural effusions, as they promote a Th1 cytokine environment through the combined effect of IL-17 plus IFN-γ stimulation of CXCL9 and CXCL10 to recruit effector T-cells [8,15]. Th17 cells have also been shown to have direct anti-proliferative and apoptosis-inducing effects toward HNSCC [16]. Studies in a mouse pancreatic cancer model showed that tumor overexpression of IL-6 increases induction of Th17 cells, reduces development of cancer and improves mouse survival [17].

Recent results suggest plasticity of CD4^+^ T-cell subpopulations with the cytokine milieu skewing the direction of differentiation. In a simplified scenario, IL-12 mediates skewing toward Th1, IL-4 towards Th2, TGF-β toward Treg, and IL-6 plus either IL-1 or TGF-β toward Th17, with the Th17 phenotype being stabilized by IL-23 [18]. Also, Foxp3^+^Treg can be reprogrammed to become Th17-like cells by IL-6 and other proinflammatory cytokines, but Treg cells can also inhibit Th17 cell expansion [8,19].

The current study showed the dynamic shifts in the immune milieu within premalignant oral lesions and when they have progressed to HNSCC, with the premalignant lesion environment consisting of inflammatory mediators and IL-17, and a sharp decline in this inflammatory environment in HNSCC. Studies further showed IL-23 to be a critical mediator derived from premalignant lesions that promotes the Th17 phenotype, but a decline in levels of IL-23 within HNSCC may be a contributor to the shift from Th17 to Treg in the HNSCC environment.

## Materials and Methods

### Patient samples

Recruitment of human subjects into this study was approved by the Institutional Review Board of record. Newly diagnosed patients with premalignant oral lesions and stage II-IV HNSCC who were being scheduled for surgery were eligible for enrollment into this study. Premalignant oral tissues and HNSCC that were surgically excised as part of the patients’ treatment plan were collected for research purposes along with peripheral blood. In addition, some of the HNSCC tissues from previously untreated patients were obtained from the Medical University of South Carolina Tissue Biorepository. While HNSCC tissue was collected from patients with stage II–IV cancer, the majority were from stage III and IV cancer patients. Normal oral tissues were also obtained from the Biorepository along with matching plasma samples. These latter tissues were deemed pathologically normal, and contained no microscopic evidence of malignant disease. Control, premalignant, and HNSCC tissues from patients were collected and homogenized by sonication. Plasma from control, premalignant lesion and HNSCC patients was separated and stored frozen until used for cytokine analyses.

### Murine model of premalignant oral lesions and HNSCC

The carcinogen 4-nitroquinoline-1-oxide (4NQO) was used to establish premalignant oral lesions and HNSCC in mice [20,21]. Two month old female C57BL/6 mice were obtained from Charles River Labs (Wilmington, MA). The 4NQO was prepared in a propylene glycol stock at 5 mg/ml and stored at 4°C until prepared for administration in the drinking water. To administer 4NQO, the stock was diluted in drinking water to 50 µg/ml. Control mice received water containing the propylene glycol diluent. The water was changed 3 times per week. To monitor development of premalignant oral lesions and HNSCC, oral cavities of 4NQO-treated mice were examined by endoscopy weekly using a Stryker 1.9 mm × 30° scope and a Stryker 1088 HD camera. Mice were sedated with inhaled isoflurane (Baxter) during the procedure. After 16 weeks of treatment, 4NQO administration was terminated. To confirm the presence of lesions versus HNSCC, tongue tissues of sample mice of each group were used for pathological examination of H&E-stained tissue sections in a blinded manner by an oral pathologist from the Center for Oral Health Research.

Tongue tissues of mice were collected and epithelial layers were stripped by enzymatic digestion at 37°C with 0.23 U Liberase (Roche) for 4 hours. As described in individual experiments, the dissociated tissue was then washed and either lysed by sonication or cultured for 36 hours prior to collection of conditioned media. Cervical lymph nodes were also harvested from C57BL/6 mice and either homogenized by sonication or dissociated via a Stomacher 80 homogenizer (Seward) set on high for 90 sec. Lysates were passed through a 40 µm cell strainer (BD Falcon) to remove debris. Viability of enzymatically dissociated cell preparations was determined by trypan blue exclusion. The media used for all cultures was RPMI-1640 medium containing 10% endotoxin-free defined fetal bovine serum, 50 µg/ml penicillin, 50 µg/ml streptomycin, 100 µg/ml neomycin, 0.02 M Hepes buffer, 5 × 10^5^ M 2mercaptoethanol and 2 mM L-glutamine.

### Cytokine measurements

The levels of IFN-γ, IL-2, IL-6 IL-17 (IL-17A) and IL-23 in tissue lysates, supernatants of dissociated tissues, or plasma were determined using a cytometric bead array cytokine kit [7]. The levels of active TGF-β in supernatants of dissociated tissues were determined using cytometric bead array flex sets according to the manufacturer’s instructions. Relative amounts of each cytokine were analyzed using FCAP Array software. All reagents used for measurement of cytokines were from BD Biosciences unless otherwise specified. Cytokine levels from both mouse and human tissues were standardized by protein levels, as determined by BCA protein assay (Pierce). Final cytokine levels in tissue were expressed as pg/100 µg of protein.

### Flow cytometric analysis of intracellular cytokine expression

In order to detect intracellular cytokines, single-cell suspensions of cervical lymph node cells or spleen cells were stimulated for 4 hours at 37°C with 50 ng/ml PMA, 1 µg/ml ionomycin, and brefeldin A solution. To block nonspecific staining, 1 × 10^6^ cells were incubated with FBS and anti-CD16/32 monoclonal antibody. They were then surface stained with PerCP-Cy5.5 CD4. Intracellular staining for PE IL-17A was performed after fixation/permeabilization with Cytofix/Cytoperm. Intracellular staining for PE-Cy7 Foxp3 (eBioscience) was performed after fixation with Foxp3 Fixation Buffer and permeabilization with Foxp3 Permeabilization Buffer (eBioscience). Positively stained cells were quantitated using a FACSCanto flow cytometer (BD Biosciences).

### Treatment of splenocytes with supernatant

In studies to delineate how premalignant lesions stimulate IL-17 levels, either unfractionated spleen cells or Th17-skewed spleen cells were incubated with fresh medium or media conditioned by cells from normal tongue epithelium, premalignant lesion or HNSCC tissues. The conditioned media used were all standardized by total protein content. For studies with Th17-polarized cells, normal CD4^+^spleen cells were cultured in polarizing media containing IL-1β (10 ng/ml), IL-21 (100 ng/ml), IL-6 (100 ng/ml), TGF-β (30 ng/ml), anti-IL-4 (10ug/ml) and anti-IFN-γ (10 µg/ml). The cells were cultured for 5 days with the addition of IL-2 (20 IU/ml) on day two. Unfractionated splenocytes or Th17-skewed CD4^+^ cells were plated at 10^5^ cells per well in 48 well dish in 500 µl of culture media along with 500 µl of media conditioned by normal tongue epithelium, premalignant lesion or HNSCC cells (prepared as described above). After 36 hours incubation, spleen cell supernatants were collected and used to measure levels of secreted IL-17 by cytokine bead array. In select experiments, the conditioned media were mixed with neutralizing antibodies to IL-23 (5 µg/ml) or recombinant IL-23 (1.5 ng/ml; R&D Systems) prior to being added to spleen cells. After 36 hours incubation, the spleen cells were incubated for an additional 4 hours with 50 ng/ml PMA, 1 µg/ml ionomycin, and brefelden A, and then surface immunostained for CD4 and intracellularly stained for Foxp3 and IL-17. Positive staining cells were analyzed by flow cytometry.

### Statistical analysis

Data were reported using the mean as a measure of central tendency ± standard error of the mean. To compare one variable condition between two groups, the 2-tailed Student's *t*-test was used. Significance was reported in the 95% confidence interval.

## Results

### Cytokine milieu of premalignant oral lesions and HNSCC, and in plasma of patients with lesions or HNSCC

Considerable attention has been given to assessing the systemic mechanisms mediating immune suppression in cancer patients such as those with HNSCC and, in particular, emphasis has been placed on measurement of immune inhibitory cytokines [22–25]. However, less is known about the intratumoral immune status and little is known about the immunological milieu in premalignant oral lesions. Our prior studies suggested an immune enhancing capacity by premalignant lesion cells. Therefore, Th1 and inflammatory cytokine levels were measured in homogenates of premalignant oral lesions that were surgically excised from patients and compared to levels in normal oral tissues and HNSCC. Also compared were cytokine levels in plasma of these same subjects.

**Figure 1:**
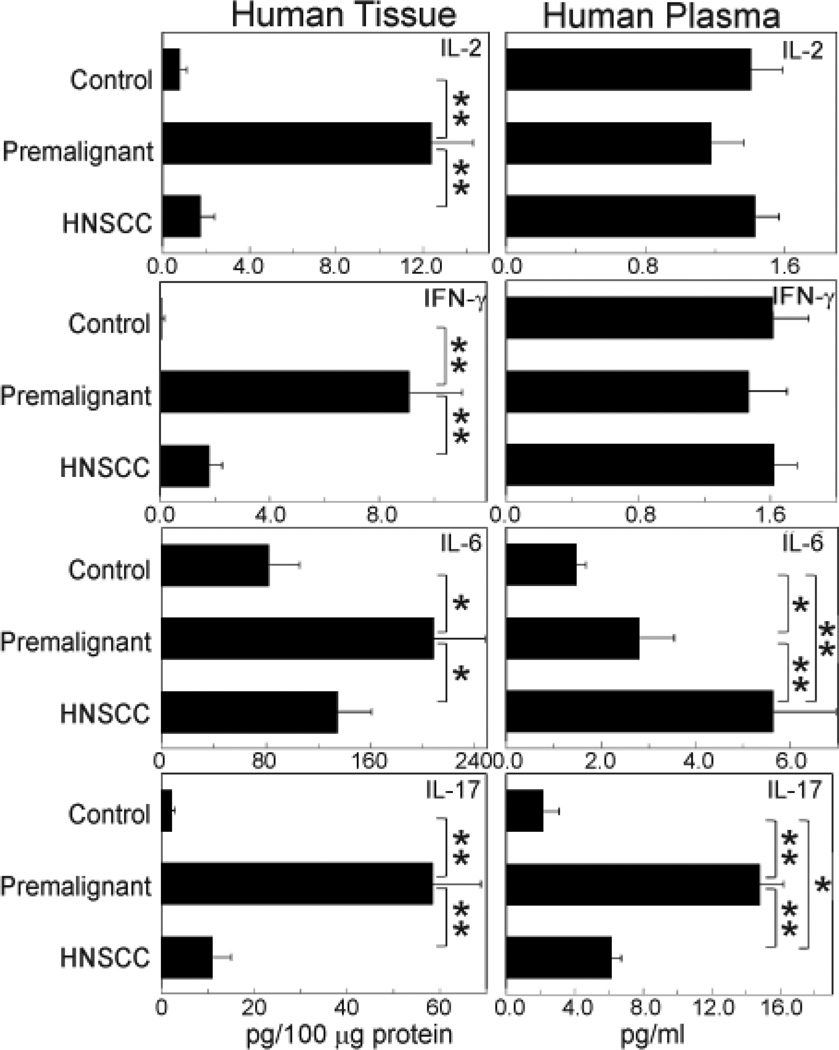
Increased Th1 and inflammatory cytokines in premalignant lesion tissue compared to levels in normal or HNSCC tissues. Normal oral tissues, premalignant lesions or HNSCC tissues were homogenized and used to measure cytokine levels using CBA. Plasma from these same patients was also analyzed for cytokine levels by CBA. Cytokine levels in tissues were standardized to levels of protein. Shown are means + SE from at least 10 patients per group. *p< 0.05; **p< 0.01.

Cytokine levels in biopsies of control normal oral tissues were low (Figure 1, left panels). This included the Th1 cytokines IL-2 and IFN-γ, as well as the inflammatory cytokines IL-6 and IL-17. In contrast, levels of each of these cytokines were significantly increased in premalignant oral lesion tissues. However, levels of these cytokines declined in HNSCC tissues and were more analogous to the levels seen in control normal tissues. The increased levels of Th1 and inflammatory cytokines seen in premalignant oral lesions did not fully translate into systemic increases. Levels of IL-2 and IFN-γ were not increased in the peripheral blood of patients with either premalignant oral lesions or HNSCC, although levels of IL-6 and IL-17 were increased in the plasma of patients with premalignant oral lesions as compared to control patients (Figure 1, right panels). Unlike what is seen in HNSCC tissues, levels of IL-6 further increased in the plasma of patients with HNSCC over the elevated levels in plasma of patients with premalignant lesions. Levels of IL-17 in plasma of HNSCC patients declined from the elevated levels in patients with premalignant lesions, akin to what is seen in the HNSCC tissues. These results suggest an immune activated and inflammatory environment within premalignant oral lesions, which subsides in HNSCC tissues. They also show the discrepancy in the cytokine phenotype between tissues and peripheral blood, suggesting localized immune skewing in premalignant oral lesions.

### Increased levels of Th1 and inflammatory cytokines in premalignant oral lesions and regional lymph nodes of mice with carcinogen-induced oral lesions and HNSCC

**Figure 2:**
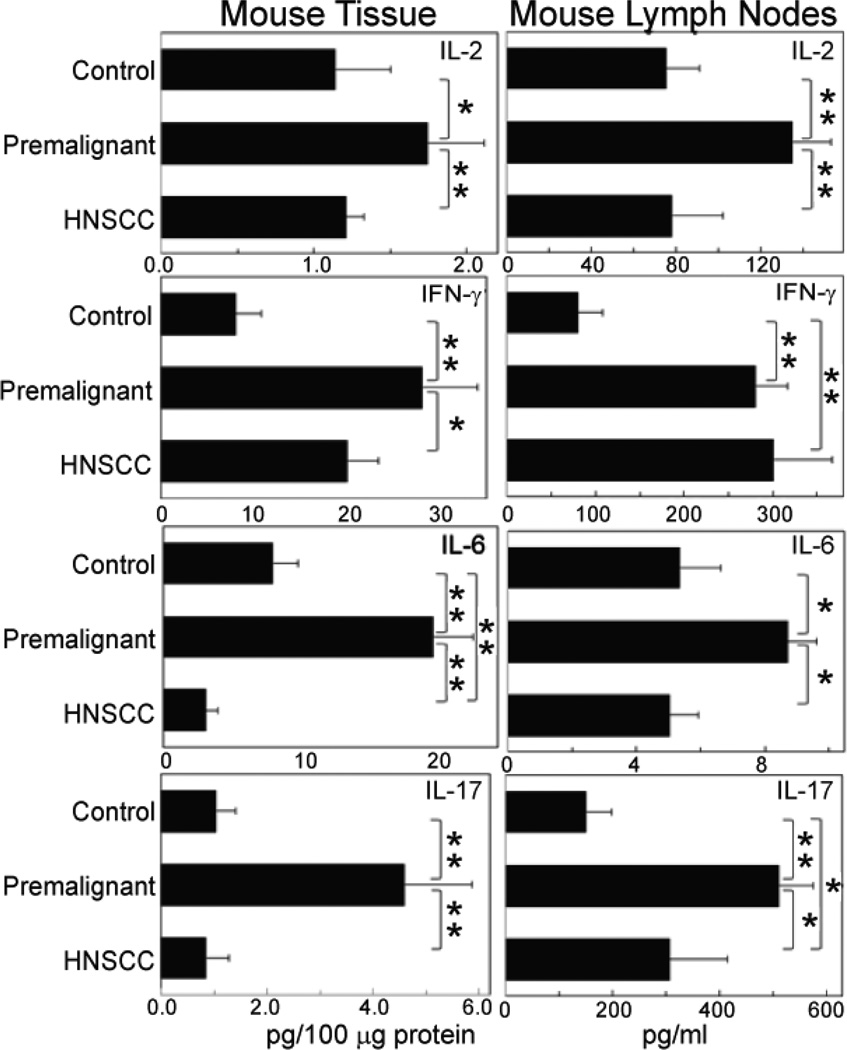
Increased Th1 and inflammatory cytokines in premalignant lesion tissues and regional lymph nodes of mice with premalignant lesions. Normal oral tissues, premalignant lesions or HNSCC tissues were homogenized and used to measure cytokine levels using CBA. Regional lymph nodes from these same mice were also homogenized by sonication and analyzed for cytokine levels by CBA. Cytokine levels in tissues were standardized to levels of protein. Shown are means + SE from at least 3 mice per group repeated 4 times. *p< 0.05; **p< 0.01.

With the goal of determining if the immune stimulated phenotype of premalignant oral lesions was also evident in regional lymph nodes, a carcinogen-induced murine model was used in which 4NQO treatment leads to the development of premalignant oral lesions primarily on the tongue, which then progress to HNSCC [20,21]. Similar to what was seen in premalignant lesions of patients, both Th1 and inflammatory cytokine levels were increased in 4NQO-induced murine premalignant oral lesions (Figure 2, left panel). These cytokine levels were also increased in the regional lymph nodes of mice with premalignant oral lesions (Figure 2, left panel). Also, similar to what was seen in human tissues, cytokine levels were reduced in HNSCC tissues of mice compared to the levels in premalignant oral lesions. Likewise, the cytokine levels in the regional lymph nodes of mice with HNSCC were reduced compared to the increased levels in lymph nodes of mice with premalignant lesions with the exception of IFNγ, which remained elevated.

**Figure 3:**
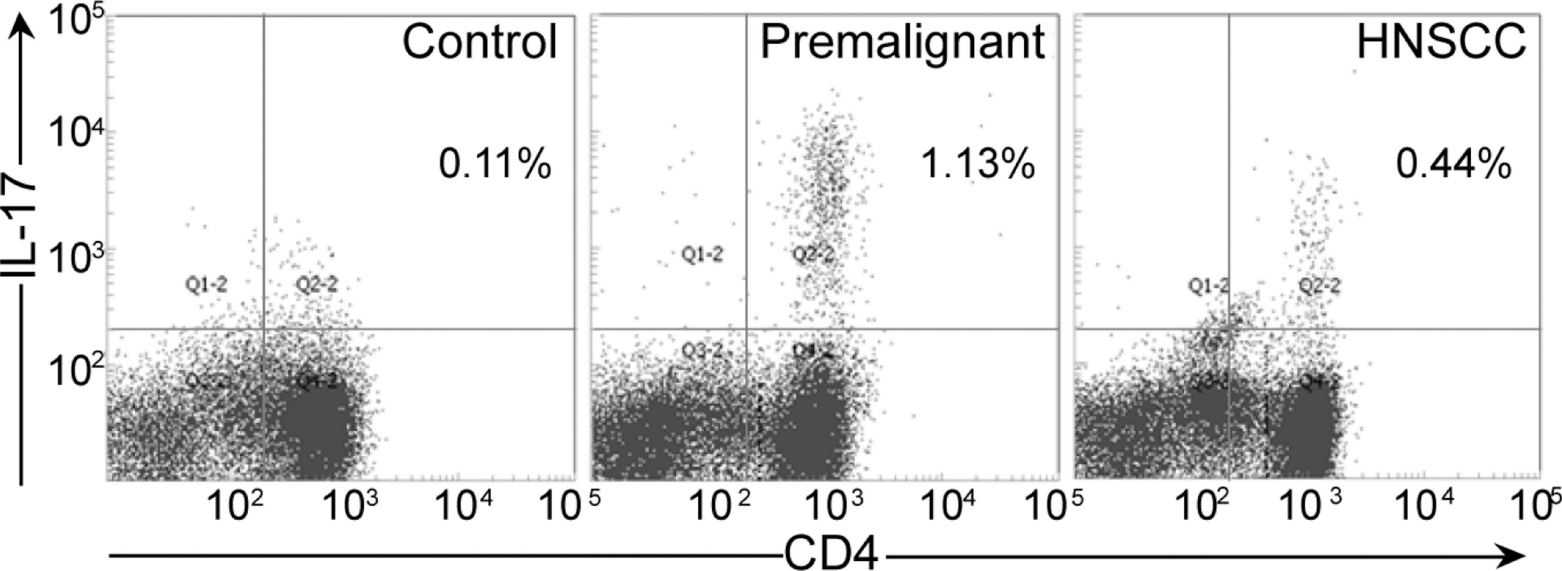
Increased levels of Th17 cells in cervical lymph nodes of mice with premalignant oral lesions. Cervical lymph nodes were collected from control mice or mice with premalignant oral lesions or HNSCC (pooled from 5 mice per group). The lymph nodes were dissociated and cells were incubated for 4 hours with PMA, ionomycin and brefeldin A. Cells were then surface immunostained for CD4, permeabilized, and immunostained for intracellular IL-17. Shown are representative scattergrams from 4 separate studies.

Because levels of IL-17 were so prominently increased in human and mouse premalignant oral lesions, as well as in plasma of patients and regional lymph nodes of mice with lesions, additional analyses of Th17 cells were conducted by measuring levels of Th17 cells. Similar to what was seen with IL-17 levels in premalignant lesion tissue homogenates, levels of IL-17-expressing CD4^+^ cells were markedly increased in lymph nodes of mice with premalignant oral lesions (1.21 ± 0.13) as compared to the low levels in lymph nodes of normal control mice (0.09 ± 0.10, p<0.001; Figure 3). Lymph nodes of mice whose lesions had progressed to HNSCC had reduced levels of Th17 cells (0.52 ± 0.05) compared to lymph nodes of mice with premalignant oral lesions (p<0.01). This coincided with the reduced levels of IL-17 shown above to be released from HNSCC tissues and from the regional lymph nodes of mice with premalignant oral lesions.

### Modulation of IL-17 production by premalignant oral lesions and HNSCC

**Figure 4:**
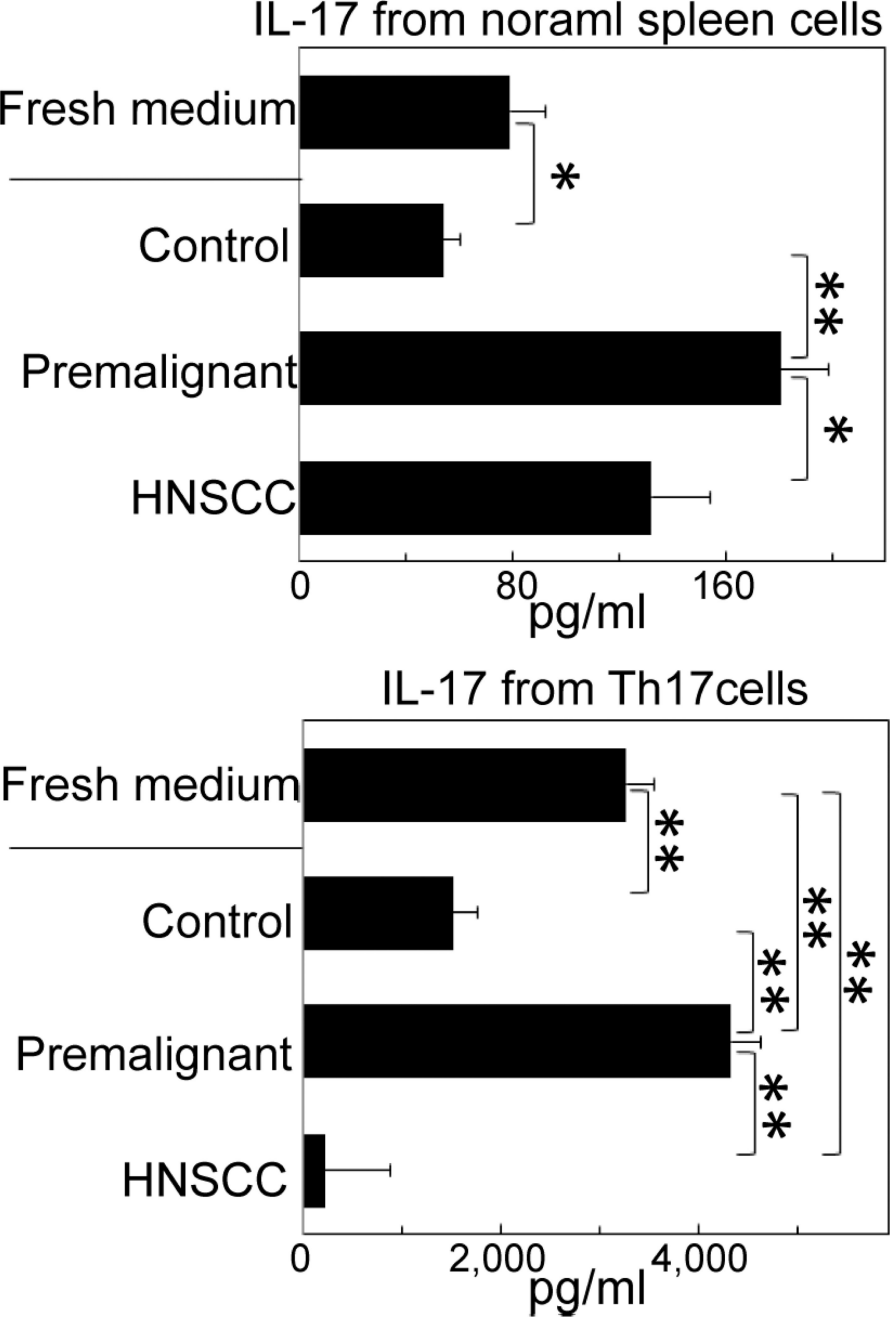
Media conditioned by premalignant oral lesions stimulates IL-17 production. Cells from enzymatically dissociated normal tongue epithelium, premalignant oral lesions and HNSCC were cultured for 36 hours. Media conditioned by these cells were then added to normal spleen cells (top graph) or Th17-skewed spleen cells (bottom graph). Levels of secreted IL-17 were measured by CBA. *p< 0.05; **p< 0.01.

Since the murine model of premalignant lesions that progress to HNSCC exhibited a similar immune phenotype as seen with human tissues, the model was used to further investigate the modulation of IL17 production by premalignant lesions and HNSCC. First determined was the impact of media conditioned by enzymatically dissociated normal tongue epithelium, premalignant oral lesions or HNSCC on normal spleen cell production of IL-17. Media conditioned by normal tongue epithelial cells was inhibitory to spleen cell production of IL-17 as compared to levels produced in the presence of fresh medium (Figure 4, top graph). In contrast, mediators from premalignant oral lesions stimulated normal spleen cell secretion of IL-17. Media from HNSCC also increased IL-17 production, but to a lesser extent than what was stimulated by premalignant oral lesions. In each of these studies, levels in the spleen cell cultures were adjusted for levels in the added conditioned media from normal, premalignant lesion or HNSCC cells, which were minimal compared to levels induced from spleen cells.

Since progression from premalignant lesions to HNSCC results in a decline in IL-17 production, studies next determined the impact of mediators from premalignant lesions and HNSCC on normal spleen cells that were Th17-polarized. After Th17 polarization, cells were incubated with fresh medium or conditioned media and their production of IL-17 was measured. Compared to levels of IL-17 produced by the Th17-polarized cells cultured without conditioned media, culture with media conditioned by from normal tongue tissue and, in particular, HNSCC suppressed IL-17 production (Figure 4, bottom graph). In contrast, media from premalignant lesions further stimulated IL-17 production.

**Figure 5:**
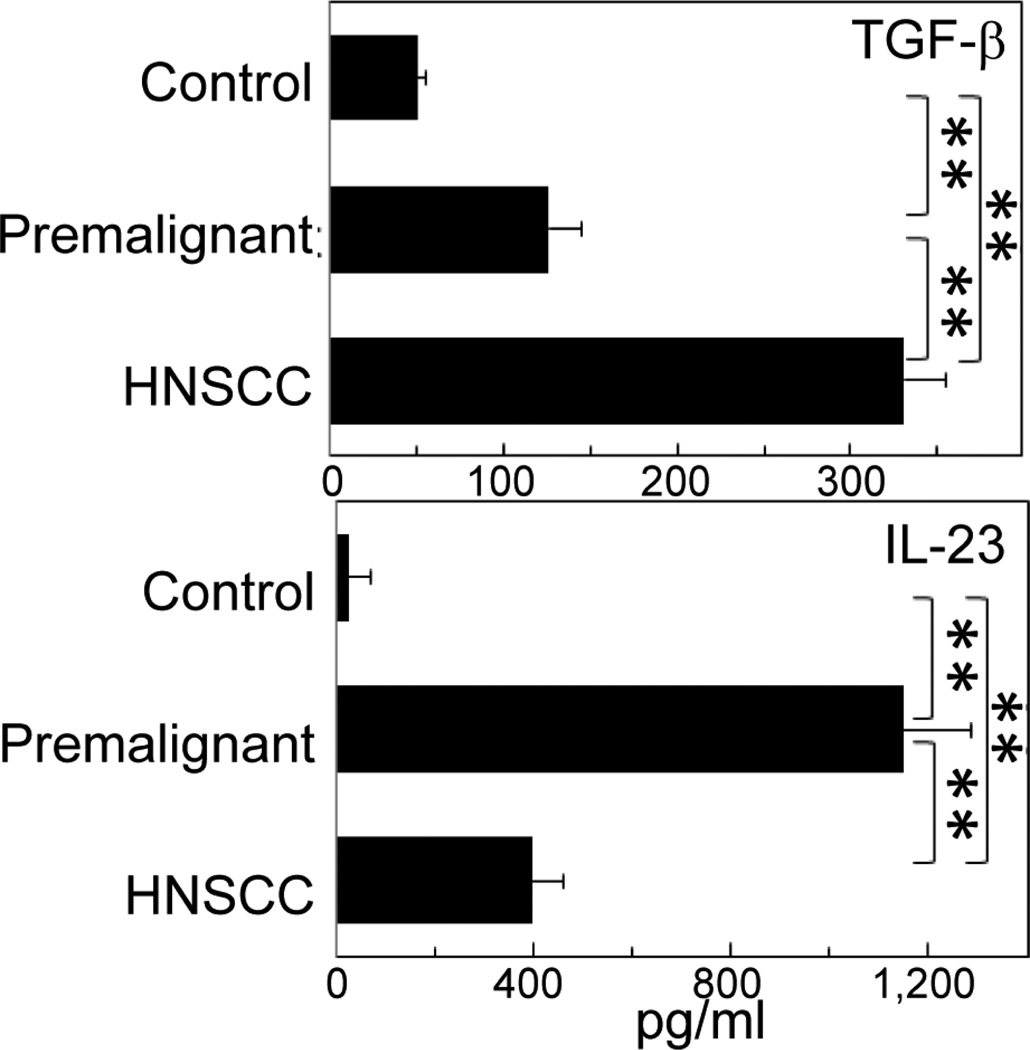
Levels of TGF-β and IL-23 produced by premalignant oral lesion cells and HSNCC. Cells from enzymatically dissociated normal tongue epithelium, premalignant oral lesions and HSNCC were cultured for 36 hours. Levels of active TGF-β and IL-23 in the conditioned media were then measured. **p< 0.01.

To determine the mechanisms by which premalignant oral lesions stimulated IL-17 production, studies first measured levels of Th17-supporting cytokines produced by premalignant lesions and HNSCC tissues. These included IL-6 plus TGFβ, which skew CD4^+^ cells toward Th17, and IL-23, which stabilizes the Th17 phenotype [18]. Levels of IL-6 (Figure 2, left panel), TGF-β and IL-23 (Figure 5) that were produced by premalignant lesion tissues were all increased in comparison to levels produced by control normal tongue epithelial tissues. HNSCC tissues produced lesser amounts of IL-6 and IL-23, but greater amounts of TGF-β than did premalignant lesions.

**Figure 6:**
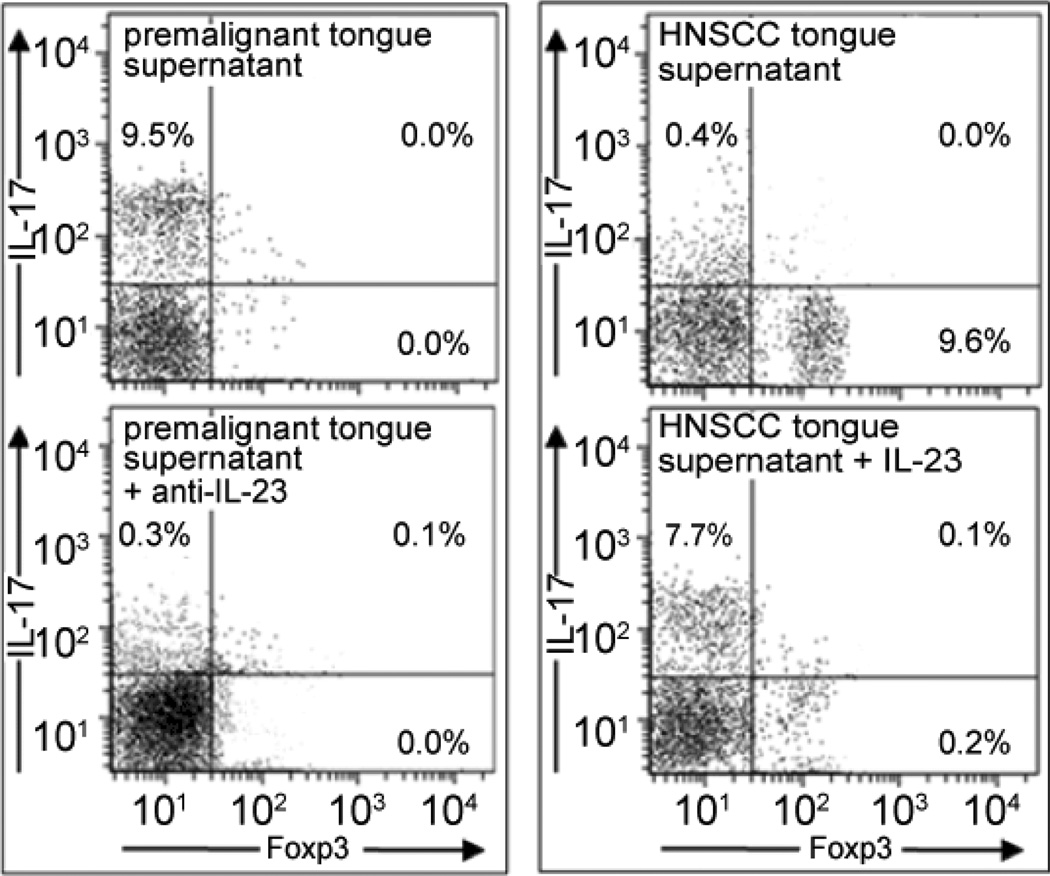
Role of IL-23 in skewing of CD4^+^ cells by premalignant oral lesions and HNSCC. Normal spleen cells were cultured with media conditioned by premalignant lesion cells that had been mixed with isotype control antibody or anti-IL-23. Alternatively, spleen cells were incubated with media conditioned by HNSCC cells plus recombinant IL-23. Shown are representative scattergrams from 4 separate studies.

The prominent production of IL-23 by premalignant oral lesion tissues led to the assessment of its contribution to the Th17 cell induction by premalignant oral lesions and the lack of Th17 induction by HNSCC. This was accomplished by neutralizing IL-23 levels in media conditioned by premalignant lesions. While IL17-expressing CD4^+^ cells were readily visible when spleen cells were incubated with media from premalignant lesions (10.06 ± 0.31), neutralization of IL-23 reduced the levels of IL-17-expressing CD4^+^ cells (0.41 ± 0.23, p<0.005; Figure 6, left graphs). Media conditioned by HNSCC tissues skewed CD4^+^ cells toward increased expression of the Treg marker Foxp3 (8.97 ± 0.63), but supplementation of HNSCC-conditioned media with recombinant IL-23 prevented the Foxp3 expression (0.24 ± 0.27, p<0.01) and instead increased IL-17 expression (7.18 ± 1.03, p<0.01; Figure 6, right graphs). This suggests the role of IL-23 in contributing to the increase in Th17/IL-17 seen in premalignant oral lesions and the decline in IL-23 in the reduced levels of Th17/IL-17 in the HNSCC milieu.

## Discussion

While studies have clearly shown that patients with HSNCC have profound immune dysfunction, very few studies have examined the immunological environment in premalignant oral lesions [4–6]. In addition, most studies with patients have focused on immune mediators, especially inhibitory mediators, in the peripheral blood rather than in the oral tissues [4,26]. Our prior studies had suggested that premalignant oral lesions are stimulatory to cytokine production, including Th1 and inflammatory cytokines [27]. Therefore, the cytokine milieu of patients and mice with premalignant oral lesions was examined, with emphasis on Th1 and inflammatory cytokines. These analyses showed low cytokine levels in normal control tongue tissues, which shifted to a prominent Th1 and inflammatory cytokine environment in premalignant lesions. However, when lesions progressed to HNSCC, levels of these cytokines declined. Furthermore, these studies showed that the cytokine phenotypes of human normal control oral tissues, premalignant oral tissues and HSNCC do not closely paralleled with plasma cytokine phenotypes. While tissues and blood specimens were collected from patients with state II-IV HNSCC, the majority were stages III and IV. Therefore, analyses of cytokine phenotypes in the present study were not stratified by HNSCC stage, but should be done in future studies.

The carcinogen-induced mouse premalignant oral lesion model provided the opportunity to not only assess cytokine shifts in tissues, which corresponded to those seen in patients, but also cytokines released from regional lymph nodes. Shifts in cytokine phenotypes in regional lymph nodes more closely paralleled shifts in tissues, contrasting to the lack of correlation between patient tissue and plasma cytokine phenotypes.

Very prominent was an increase in IL-17 in premalignant oral lesion tissues of patients and mice, as well as in plasma of patients and regional lymph nodes of mice with premalignant oral lesions. Studies to assess possible mechanisms underlying this increase in IL-17 showed IL-23 to be a critical mediator derived from premalignant lesions that promotes the Th17 phenotype. Furthermore, the reduction in production of IL-23 and the increase in TGF-β production by HNSCC are likely contributors to the shift from Th17 to Treg in the HNSCC environment.

Since their discovery, IL-17-producing cells have risen to prominence in studies of autoimmune disease, allergies, inflammation andmicrobial infections [28–31]. While their role in the pathogenesis of these conditions is well defined, their function in the context of tumor immunology remains controversial. Similar to our findings, others have shown Th17 cells to be in increased levels early in gastric disease and ovarian cancer, with a shift from Th17 to Treg cells in advanced disease [8,9]. In early stage ovarian cancer and malignant pleural effusions, Th17 cell presence has been associated with improved prognosis as they promote a Th1 cytokine environment through enhanced recruitment of effector T-cells [8,15]. *In vitro* studies have shown a direct anti-proliferative and apoptosis-inducing effect of Th17 cells toward HNSCC [16]. Studies in a mouse pancreatic cancer model showed that implantation of tumor cells engineered to overexpress IL-6 results in induction of Th17 cells, reduced development of cancer and improved mouse survival [17]. In contrast to studies showing anti-tumor effects of a Th17 phenotype, other studies have shown that the presence of Th17 cells increases expression of pro-angiogenic mediators and, in particular, VEGF [32]. This could increase the likelihood of cancer metastasis, although the proangiogenic and metastasis-promoting effects of Th17 cells have not been extensively tested. There is a body of literature implicating Th17 cell-associated inflammation as being a pro-tumorigenic environment [13,14]. Thus, the role of Th17 cells and their contribution or deterrence to tumor progression is paradoxical and not well defined, with the literature being divided. It is possible that the cytokinestimulated phenotype in premalignant oral lesions and regional lymph nodes is a reflection of a failing immune attempt at preventing malignant progression.

In the studies currently described, incubation of normal spleen cells with media conditioned by premalignant lesions increased IL-17 levels over levels produced following incubation with media from normal tongue epithelium. Incubation with media from HNSCC induced only a minor increase in IL-17. Unexpected was the impact of these conditioned media on IL-17 production by Th17-skewed cells. Mediators from normal tongue epithelium were highly inhibitory to IL-17 productions, although the identity of the inhibitory mediators has not yet been determined. In contrast, media from premalignant lesions further enhance IL-17 production by Th17-skewed cells. The demonstration that premalignant lesion-induced skewing toward Th17 is dependent on IL-23 and the increased production of IL-23 by premalignant lesion tissues implicate IL-23 as a likely candidate by which premalignant lesions skew CD4^+^ cells toward Th17 cells. Contrasting with the prominent skewing by premalignant lesions toward the Th17 phenotype, mediators from HNSCC instead skewed CD4^+^ cells toward a Treg phenotype. This skewing toward Treg was preventable by supplementation with IL-23, which could suggest that the Treg skewing by HNSCC is associated with their reduced production of IL-23 and increased production of TGF-β. Future studies designed to block the premalignant lesion-derived mediators that promote the inflammatory and Th17 phenotype could resolve if the contribution of this phenotype is to accelerate or to prevent progression of premalignant lesions to oral cancer.
